# The relationship between number of primary health care visits and hospitalisations: evidence from linked clinic and hospital data for remote Indigenous Australians

**DOI:** 10.1186/1472-6963-13-466

**Published:** 2013-11-06

**Authors:** Yuejen Zhao, Jo Wright, Steven Guthridge, Paul Lawton

**Affiliations:** 1 Department of Health, PO Box 40596, Casaurina, NT 0811, Australia; 2 Menzies School of Health Research, PO Box 41096, Casaurina, NT 0811, Australia

**Keywords:** Access, Acute inpatient care, Administrative data uses, Primary care, Rural health

## Abstract

**Background:**

Primary health care (PHC) is widely regarded as essential for preventing and treating ill health. However, the evidence on whether improved PHC reduces hospitalisations has been mixed. This study examines the relationship between PHC and hospital inpatient care in a population with high health need, high rates of hospitalisation and relatively poor PHC access.

**Methods:**

The cross-sectional study used linked individual level PHC visit and hospitalisation data for 52 739 Indigenous residents from 54 remote communities in the Northern Territory of Australia between 1 July 2007 and 30 June 2011. The association between PHC visits and hospitalisations was modelled using simple and spline quadratic regression for key demographics and disease groups including potentially avoidable hospitalisations.

**Results:**

At the aggregate level, the average annual number of PHC visits per person had a U-shaped association with hospitalisations. For all conditions combined, there was an inverse association between PHC visits and hospitalisations for people with less than four clinic visits per year, but a positive association for those visiting the clinic four times or more. For patients with diabetes, ischaemic heart disease or renal disease, the minimum level of hospitalisation was found when there was 20–30 PHC visits a year, and for children with otitis media and dental conditions, 5–8 visits a year.

**Conclusions:**

The results of this study demonstrate a U-shape relationship between PHC visits and hospitalisations. Under the conditions of remote Indigenous Australians, there may be an optimal level of PHC at which hospitalisations are at a minimum. The authors propose that the effectiveness of a health system may hinge on a refined balance, rather than a straight-line relationship between primary health care and tertiary care.

## Background

Primary health care (PHC) is widely regarded as an essential community service with a role to prevent and treat ill health or, once a condition is established, to maintain optimal health. PHC is also the major entry point to the total health system [1]. While a common perception is that improved access to PHC can reduce hospitalisations, the evidence has been mixed. Some studies suggest an inverse association between PHC and hospitalisation [2-4], with increased number of PHC visits linked to savings in hospitals and improvements in health outcomes [5,6]. By contrast, other studies have reported a positive association, with improved PHC access leading to increased hospital referrals [7,8]. A third group of studies have reported no association between the two types of care [9,10]. Despite the difference in outcome between the three groups of studies, what they have in common is that they all explicitly or implicitly have assumed a straight-line relationship between PHC and hospital care when the actual relation may be curvilinear or nonlinear. Large scale empirical studies are lacking in this area.

In Australia, PHC is funded through the Medicare Benefits Schedule (MBS) under a policy of universal access. However in the Indigenous population of the Northern Territory (NT), a population with high health need [11], MBS per capita payments are less than 50% of their non-Indigenous peers [12]. This reduced PHC access coincides with a hospitalisation rate 7.7 times that of other Australians [13], which raises the possibility that the lower access to PHC services may be, in part, responsible for higher hospitalisation rates. Poor health outcomes for Indigenous Australians is of national concern, highlighted by the recent agreement by the Council of Australian Governments to close the Indigenous gap in health outcomes [14]. At the same time there are efforts in Australia to curb escalating government health expenditure by reducing hospitalisations [13]. Improved PHC is considered to be the key to both challenges. Access to PHC may be measured in relation to the availability, utilisation or outcomes of services [15]. This study explores the relationship between PHC utilisation and hospitalisations in a population with high health need, high hospitalisation rates and poor PHC access.

The NT is a federal territory of Australia, occupying much of the centre and top end of the continent. According to the Australian Statistical Geography Standard, 99.8% of the NT is classified as either a Remote or Very Remote (hereafter called remote) area [16]. The remote area of the NT, equivalent in size to five United Kingdoms, has approximately 40 medical practitioners providing PHC for about 51 000 Indigenous residents [17], 80% of the total NT Indigenous population (about 64 000 in 2006). The majority of PHC providers in remote areas are nurses (approximately 400) and Aboriginal health workers (200) [18], employed by either the NT Department of Health (DOH) or Australian Government funded Aboriginal health services. Few PHC services are provided by allied health professionals. Hospital services are provided by a network of five public hospitals (Alice Springs Hospital, Gove District Hospital, Katherine Hospital, Royal Darwin Hospital and Tennant Creek Hospital). The median distance from a remote Indigenous community to the nearest hospital is 275 kilometres (kms), ranging from 87 to 700 kms.

The aim of this study was to examine the association between the numbers of PHC visits and public hospital admissions among Indigenous residents of remote communities. The analysis included a breakdown by key demographics and common conditions such as adults with hypertension, diabetes, ischaemic heart disease (IHD), kidney diseases, and chronic obstructive pulmonary disease (COPD), and children with gastroenteritis, respiratory infection, malnutrition, otitis media, dental caries and rheumatic heart disease (RHD). To the best of our knowledge, no previous studies have investigated the PHC-hospital association in a remote Indigenous setting.

## Methods

The relationship between numbers of PHC visits and hospitalisations was assessed by using individual-level cross-sectional data. Participants were included if, during the study period, they had either a clinic visit or public hospital admission with a residential address of one of 54 NT remote Indigenous communities or associated outstations. In this study, a PHC visit was defined as a face-to-face encounter between a patient and physician, nurse, Aboriginal health worker or other PHC provider. The PHC services are routinely recorded in the centralised Primary Care Information System (PCIS). Hospitalisation data were gathered from all five NT public hospitals in the centralised hospital information system (Caresys). The study period was four years, from 1 July 2007 to 30 June 2011. The study carried out deterministic linkage of individual-level clinic and hospital data using Hospital Registration Number (HRN). Shared by PCIS and Caresys, the HRN is a unique patient identifier developed and used in the NT for more than 20 years and has been demonstrated to be highly reliable with accuracy rates for Indigenous status 98%, sex 99%, year of birth 91% and locality 88% [19]. The HRN has also been used for eHealth records so that health care providers, including non-DOH providers, can retrieve clinical information on shared clients [20].

Disease groups were defined using the International Classification of Primary Care (ICPC) [21] and the Australian Refined Diagnosis Related Groups (AR-DRG) (see Table 1) [22]. Clinic records with an invalid ICPC code or ICPC component code 67 (referral to hospital or specialist) were excluded. Age was derived using date of birth and date of first contact. The International Classification of Diseases and Related Health Problems, 10^th^ Revision, Australian Modification was used to identify potentially avoidable hospitalisations (PAH), applied to principal and secondary diagnoses and procedure codes [23]. PAHs, also called ambulatory care sensitive conditions, are believed to be responsive to timely PHC interventions [23].

**Table 1:** List of disease groups and definitions

**Disease group**	**Primary care ICPC codes**	**Hospital AR-DRG codes**
**Diabetes**	F83, T87, T88, T89, T90	F11A, F11B, F13Z, K01Z, K60A, K60B
**Ischaemic heart disease**	K74, K75, K76, K89	F08A, F08B, F14A, F14B, F14C, F12Z, F01A, F01B, F02Z, F66A, F66B, F74Z, F72A, F72B, F05A, F05B, F06A, F06B, F17Z, F18Z
**COPD**	R91, R95	E65A, E65B, E69A, E69B, E69C
**Renal disease**	U88, U90, U95	L65A, L65B, L67A, L67B, L67C, A09A, A09B, L02A, L02B, L60A, L60B, L60C, L61Z
**Hypertension**	F83, K85, K86, K87	F67A, F67B
**Rheumatic heart disease**	K71, K83, L88	F69A, F69B, I66A, I66B, F75A, F75B, F75C, F03Z, F04A, F04B
**Respiratory infection (age < 15 years)**	R05, R71, R74, R78, R79, R81, R83	E62A, E62B, E62C, E69A, E69B, E69C, E70A, E70B
**Gastroenteritis (age < 15 years)**	D11, D70, D73, D94	G67A, G67B, G68A, G68B
**Malnutrition (age < 15 years)**	T10, T91, B80, B82	K61Z, Q61A, Q61B, Q61C
**Otitis media (age < 15 years)**	H70, H71, H72, H73, H74	D63A, D63B
**Dental caries (age < 15 years)**	D19, D82	D40Z, D67Z

Notes: AR-DRG = Australian Refined Diagnosis Related Groups; COPD = chronic obstructive pulmonary disease; ICPC = International Classification of Primary Care.

The average numbers of PHC visits and hospitalisations per person per year (person-year) and average length of hospital stay were analysed by age group, sex and selected disease groups to summarise the relationship between PHC and hospital care. A bubble diagram was applied to depict three-dimensional information [24] with bubble area representing population size. The PHC-hospital relationship was further explored with simple and spline quadratic regressions [25]. The spline quadratic model glues two simple quadratic models together through a free knot at the vertex. The spline quadratic model fit the data better than the simple quadratic model, because of the additional parameters introduced. The goodness-of-fit of the models were assessed using Pearson’s chi-square test [26]. The modelling was performed in Stata/IC 12.0 software and MS Excel. To improve goodness-of-fit and robustness, the modelling truncated individuals with clinic visits greater than 200 times over the four-year study period (1.43% of total patients). Sensitivity analysis was undertaken to test the alternative assumptions, such as free or fixed knot of spline quadratic models, different age groupings and truncating criteria of PHC visits. Simple quadratic models were used for comparing the demographic and disease-specific relationships.

This study was approved by the Human Research Ethics Committee of the DOH and Menzies School of Health Research (Reference number: HREC-2012-01723).

## Results

**Figure 1:**
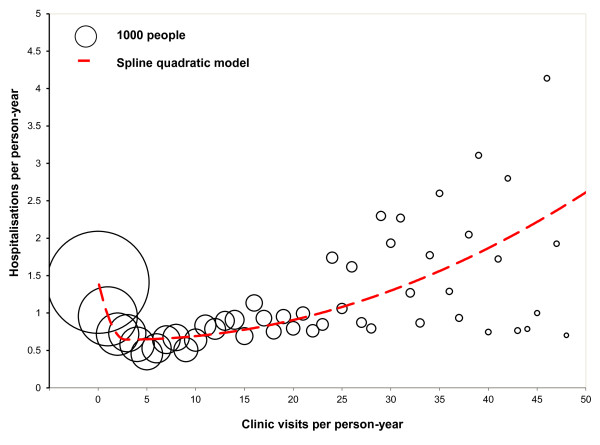
**Average hospitalisations per person-year by average annual clinic visits for remote Indigenous patients, with a spline quadratic model, Northern Territory, Australia, 2007–2011.** Note: The size of bubbles denotes the number of patients.

**Figure 2:**
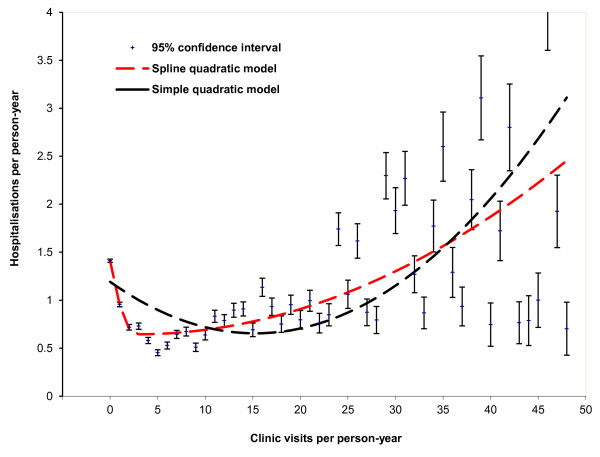
Average hospitalisations per person-year by average annual clinic visits for remote Indigenous patients with 95% confidence intervals, comparing two quadratic models, Northern Territory, Australia, 2007–2011.

There were 1 296 977 PHC visits and 216 819 public hospital admissions included in the study. There was a total of 52 739 patients in the linked data (48% male, 52% female), who were recorded as residing in the catchment areas of the 54 DOH clinics. This indicates that the majority (82%) of the NT Indigenous population had a remote area address and used a DOH service, at least once, during the study period. Of the total number of patients, 35% were between 0 and 14 years of age, 42% 15–39, 18% 40–59 and 5% aged 60 years and over. Through the HRN linkage, 35% of patients, 69% of clinic visits and 56% of hospitalisations were linked between the clinic and hospital data. The average number of PHC visits was 6.1 per person-year, and the average number of hospitalisations was 1.0 per person-year. At the aggregate level, 5.1% of patients were recorded as having diabetes, 3.4% hypertension, 3.3% renal disease, and 3.0% as having IHD or COPD. Among children aged 0–14 years, 38.3% experienced a respiratory infection, 29.5% otitis media, 18.0% gastroenteritis, 12.6% dental caries, 7.8% malnutrition and 1.7% had RHD. Table 2 provides the average hospitalisations per person-year and average length of hospital stay (in days) by the average PHC visits with 95% confidence intervals. Over one-third (37%) of patients visited a PHC clinic less than once a year, on average, during the four years. The average number of hospitalisations was 1.41 per person-year for people with less than one PHC visit per year, significantly higher than those with more PHC visits (P < 0.05). The average hospitalisations decreased with increasing PHC visits to a minimum of 0.45 admissions per person-year when the patients visited a clinic 5 times a year. Hospitalisations then increased with increasing PHC visits for those having more than 5 visits a year. For those who visited the clinics 12 times a year and more, the hospitalisation rate was 1.17 per Person-year. Hospitalisation rates appeared to be associated with PHC visits in a nonlinear fashion, and the relationship between PHC visits and hospitalisations appeared a U-shape (Figure 1). This U-shaped association was also evident for hospital bed-day utilisation (Table 2). Patients with zero PHC visits stayed in hospital 2.52 days on average, whereas those with four PHC visits stayed 1.95 days on average and those with 12 PHC visits and more stayed an average of 3.29 days. The spline quadratic regression model (see the dashed line in Figure 1) indicates that there was an inverse association between PHC visits and hospitalisations for people with less than four clinic visits per year, but a positive association for those visiting the clinics more than four times a year. Figure 2 demonstrates that the distribution of the association became increasingly heterogeneous, and the variability of hospitalisation rates tended to increase with PHC visits, when the number of PHC visits was more than 15 times a year. Figure 2 also indicates that the spline quadratic regression model (dashed curve) had more flexibility and capacity to model complicated data than the simple quadratic model (solid curve).

**Table 2:** Average hospitalisations per person-year and average length of hospital stay by frequency of clinic visits, Northern Territory, 2007-2011

**Annual visits**	**Number of patients (%)**	**Annual hospitalisations (95% CI)**	**Average length of stay (days) (95% CI)**
**0**	19690 (37%)	1.41 (1.28-1.54)	2.52 (2.51-2.53)
**1**	6600 (13%)	0.96 (0.75-1.16)	2.36 (2.33-2.39)
**2**	3393 (6%)	0.72 (0.49-0.94)	2.03 (2.00-2.06)
**3**	2609 (5%)	0.73 (0.48-0.98)	2.13 (2.09-2.17)
**4**	2245 (4%)	0.58 (0.35-0.81)	1.95 (1.91-1.99)
**5**	1892 (4%)	0.45 (0.30-0.61)	2.17 (2.12-2.22)
**6**	1609 (3%)	0.53 (0.32-0.73)	2.70 (2.62-2.78)
**7**	1449 (3%)	0.64 (0.40-0.89)	2.13 (2.08-2.19)
**8**	1318 (2%)	0.67 (0.38-0.97)	2.22 (2.16-2.28)
**9**	1084 (2%)	0.51 (0.41-0.61)	2.04 (1.98-2.10)
**10**	939 (2%)	0.64 (0.44-0.83)	3.37 (3.27-3.48)
**11**	842 (2%)	0.83 (0.48-1.19)	2.85 (2.77-2.94)
**12+**	9069 (17%)	1.17 (1.03-1.31)	3.29 (3.28-3.30)
**Total**	52739 (100%)	1.06 (0.99-1.12)	2.70 (2.69-2.71)

Note: CI = confidence interval.

Table 3 provides the quadratic vertex estimates of PHC visits corresponding to the minimum level of hospitalisations, estimated by simple and spline quadratic regressions. As the results in Table 3 demonstrate, the PHC levels associated with the lowest hospitalisation rate for the overall population were detected to be 4 and 15 visits per person-year by the spline and simple quadratic models respectively. PAH levels were minimised when providing 2–17 clinic visits per person-year. Of the adults with the chronic diseases, hospitalisations were minimised for those who were provided with 20–30 PHC visits. The goodness-of-fit statistic shows that the spline model fit the data better than the simple quadratic model, and the model fit the data by key demographics and child health conditions better than adult chronic conditions (P < 0.01), indicating the U-shape association is more apparent in the general population and child health conditions when the sample size is greater.

**Table 3:** Estimates of the average number of annual clinic visits associated with minimum hospitalisations for demographic and disease groups, using two quadratic models, Northern Territory, 2007-2011

**Group**	**Optimal clinic visits**		**Goodness-of-fit (χ**^**2**^**)**	
	**Spline model**	**Quadratic model**	**Spline model**	**Quadratic model**
**Total**	4	15	1284.3*	4381.5*
**Female**	5	16	1322.4*	2360.0*
**Age 40+ years**	9	24	2117.3*	2542.2*
**PAH**	2	17	1477.2*	3127.3*
***Adult chronic diseases***				
**Diabetes**	23	28	2929.7^#^	4599.1^#^
**IHD**	27	28	3169.3^#^	4066.8^#^
**COPD**	22	20	2998.0^#^	3586.3^#^
**Renal disease**	30	29	2848.8^#^	7696.8^#^
**Hypertension**	20	25	2913.1^#^	5292.8^#^
***Child health conditions (age < 15)***				
**Respiratory infection**	3	12	44.4*	532.1*
**Rheumatic heart disease**	6	20	30.6*	35.9*
**Gastroenteritis**	4	17	46.0*	162.9*
**Malnutrition**	2	11	49.8*	114.3*
**Dental**	5	8	28.3*	112.0*
**Otitis media**	5	8	91.6*	336.9*

Notes: ^#^ P < 0.01; * P > 0.95; COPD = chronic obstructive pulmonary disease; PAH = potentially avoidable hospitalisation [23]; χ^2^ = chi-square.

**Figure 3:**
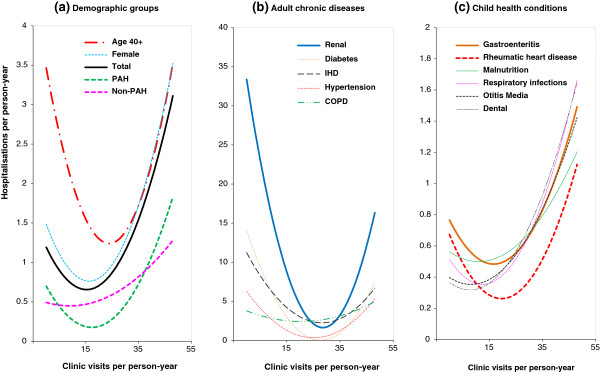
**Average hospitalisations per person-year by average annual clinic visits for (a) demographic groups, (b) adult chronic diseases and (c) child health conditions for remote Indigenous patients, using simple quadratic models, Northern Territory, Australia, 2007–2011.** Note: COPD = chronic obstructive pulmonary disease; IHD = ischaemic heart disease; PAH = potentially avoidable hospitalisation.

Figure 3 uses simple quadratic regression lines to compare the impacts of key demographics, chronic diseases and child health conditions on the PHC-hospital relationship. Inspecting panel a in Figure 3, we see that PAH (short green dashes) decreased from 0.7 to 0.2 hospitalisations per person-year when PHC visits increased from 0 to 15 visits annually. In other words, at least two-thirds of PAHs may potentially be avoided by providing adequate levels of PHC. By comparing with the total hospitalisations (solid black curve), this difference was equivalent to a reduction of PAHs from 59% to 28% of the total hospitalisations. In contrast, the curve for non-PAH was rather flat (pink dashes in panel a), and generally increased with PHC visits. Panel a in Figure 3 also compares the PHC-hospital relations by key demographics. The PHC visits associated with the minimum level hospitalisation was slightly greater in females (5–16 visits per person-year) and much greater in people aged 40 years and over (9–24) (Table 3 and panel a, Figure 3). Patients with renal disease, diabetes, hypertension and IHD showed a clearer effect of U-curve than COPD (panel b, Figure 3). The U-curve effects were more pronounced for children with gastroenteritis, respiratory infection and RHD than the other three conditions (panel c). It is also noteworthy that children with 5–8 clinic visits a year for otitis media and dental conditions, and 6–20 visits a year for RHD had the minimum level of hospitalisations (Table 3). For clarity, spline quadratic models and 95% confidence intervals for demographics, chronic diseases and child health conditions were omitted from Figure 3. Sensitivity analysis reveals that including truncated outliers of excessive clinic visits (200+) did not significantly alter the results but reduced overall fit. Further analysis revealed that these truncated patients were more likely to have one or more chronic conditions (50.1% diabetes, 20.5% IHD, 23.0% renal disease, compared with 5.1%, 3.0% and 3.3% in the total respectively), and more likely to be older (23.6% aged 60 and over vs 5.3%) and female (64.5% vs 52.4%). Removal of same day haemodialysis from the analysis resulted in reduction of the hospitalisations due to renal disease, but the U-curve effect remained (data not shown).

## Discussion

Understanding the association between PHC and hospital care is important for the efficient use of health care resources [4], especially in rural and remote settings. This study demonstrates that too little PHC may lead to an excess of both hospitalisations and length of hospital stay, but so does too much, with people who receive either less or more than the optimal level of PHC having a marked increase in number and length of hospitalisations. The U-shape relationship is also consistent across various population sub-groups including: people over 40, females, and those with chronic conditions. These findings add to the evidence that improved access to PHC may prevent hospitalisations, improve health outcomes and lower health care costs [3,6]. Few studies have attempted to estimate an optimal level of medical care. Ledwidge and colleagues [27] reported the required number of clinic visits was two per month to prevent hospitalisations for heart failure, while others have reported that an average of 4–5 visits a year was required to develop a sufficient knowledge base for health care continuity [28]. This study supports an argument that providing an optimal level of PHC in remote Indigenous communities may reduce hospitalisations, although the optimal levels of PHC service may vary with age, gender and disease.

The U-shaped distribution provides evidence for a nonlinear association between PHC activity and hospitalisation, and draws together the contradictory results of previous studies [3,8,10]. A similar nonlinear pattern was also reported for the effect of distance on hospitalisation [29]. Lin and colleagues found the lowest hospitalisation rates among residents living between 35 and 50 kms from a hospital. Living either closer to (<35 kms) or further from (>50 kms) a hospital was associated with higher hospitalisation rates. In this study the communities were all located far from a hospital (≥87 kms). There may be a number of reasons that the PHC-hospital association varies with the level of PHC. Low levels of PHC may lead to increased false negative and delayed diagnoses, acute evacuation and hospitalisation [30,31]. Under this circumstance, investment in PHC can improve prompt diagnosis and treatment that may avert or postpone the need for hospital care. This inverse relationship is consistent with the majority of literature [4-6], especially those studies undertaken in PHC shortage areas. Patients receiving PHC beyond the optimal level may be at the more severe end of clinical spectrum and require both more PHC and hospital services. In this case, PHC is not a substitute for hospital care, but a complement [32]. It is also possible that a portion of the extra hospitalisations are a result of increased false positive diagnoses arising from the increased PHC contacts, leading to more hospital referrals. This possibility has been recognised in previous studies [7,8]. Planned consultations and elective admissions tend to be positively correlated and in these cases, an expansion of PHC services may not reduce hospitalisations. There is increased heterogeneity in the distribution of results among the frequent PHC users at the right upper part of the U-curve, a group of patients with high levels of both PHC and hospital services. For this group, PHC may be insufficient for complex needs and there may be the opportunity to reduce both PHC and hospitalisations through specialised case management [33].

Adequate PHC is considered to be essential [1]. The current level of access to PHC for Indigenous residents in remote areas is inadequate compared with the national average, even before consideration of the greater health need [11,12] and the need for culturally appropriate services [34]. Residents in PHC shortage areas are more likely to experience hospitalisations, and optimising PHC service levels can improve health and reduce health inequality [2]. PHC plays an important role in improving Indigenous health outcomes and reducing the adverse effects of health inequity, because PHC is cost-efficient for prevalent conditions [35]. Hypertension, chronic kidney disease, diabetes, asthma, IHD, COPD, pneumonia and urinary tract infections are common presenting problems at the NT remote clinics. Unless they progress to serious complications, such conditions are more appropriately managed by prompt interventions in PHC settings than hospitalisation.

Strengths and limitations: The strengths of this study are that for the first time, to our knowledge, the study demonstrates the U-shape association between PHC and hospital care. The methodological limitations of previous studies have been overcome by using quadratic regression models and examining routinely collected large scale service data. The spline quadratic model fits the aggregate data better than the simple quadratic model, but does so at the expense of robustness and parsimony. The spline regression model provides the advantage that, being more sensitive to the data, it is more useful when deriving vertex values. On the other hand, the simple quadratic model is more robust and more readily interpretable, making it useful for comparisons within a family of U-curves. There are also a number of limitations. Firstly, the strength of the evidence is limited by the reliability of clinic and hospital data. There is an ongoing program of consolidation and validation to maintain the quality of HRN, with the accuracy of patient demographic information in public hospital records recently reported as around 95% [19]. There have also been clinical audits, which have confirmed the quality of data collections [19,36]. Deterministic linkage is simple but considered a more reliable linkage strategy, when coding errors of HRN are minimal [37]. Secondly, this study did not control individual level variations and potential confounders such as types of PHC, professions of PHC providers and distance to hospital. More research is needed to further explore this topic. Multilevel analysis and multivariate adaptive regression splines may be a useful tool [38]. Thirdly, the study did not include people who were not recorded with either a clinic visit or hospitalisation during the study period, however the total study population was similar to the Indigenous resident population in the selected remote areas [17]. Additionally, PHC data were incomplete due to high population mobility, unclear clinic catchments and the availability of alternate non-DOH PHC services. While this incompleteness may lead to an underestimate of the optimal number PHC services for the population, it is unlikely to change the general pattern of the U-curve association between PHC and hospitalisations. Finally, this study is neither longitudinal nor experimental, which limits the extent to which a causal relation can be drawn and generalised. Continued recording of clinical events and the maintenance of clinical quality audits will facilitate the opportunity for longitudinal and experimental studies for this topic in the future.

## Conclusions

An effective PHC and hospital interface is important to achieve optimal health outcomes and cost-efficiency of the health care system. The results of this study demonstrate a U-shape relationship between PHC visits and hospitalisations, and support an argument that remote Indigenous people in Australia may have fewer hospitalisations with an appropriate level of primary care. The results suggest that the effectiveness of a health system is not simply a straight-line relationship in which “more PHC is better”, but instead hinges on a refined balance between optimal primary health care and tertiary care.

## Abbreviations

AR-DRG: The Australian Refined Diagnosis Related Groups; COPD: Chronic obstructive pulmonary disease; DOH: The NT department of health; HRN: Hospital registration number; ICPC: International Classification of Primary Care; IHD: Ischemic heart disease; kms: kilometers; MBS: Medicare Benefits Schedule; NT: Northern Territory of Australia; PAH: Potentially avoidable hospitalisations; PCIS: Primary Care Information System; PHC: Primary health care; RHD: Rheumatic heart disease.

## Competing interests

The authors declare that they have no competing interests.

## Authors’ contributions

JW and SG helped to design this study, and facilitated data access; YZ conducted the analysis and interpretation of the data with the assistance by SG, JW and PL. YZ and SG lead the writing of this report. PL provided constructive insights to improve the report. All authors read and approved the final manuscript.

## Pre-publication history

The pre-publication history for this paper can be accessed here:

http://www.biomedcentral.com/1472-6963/13/466/prepub
